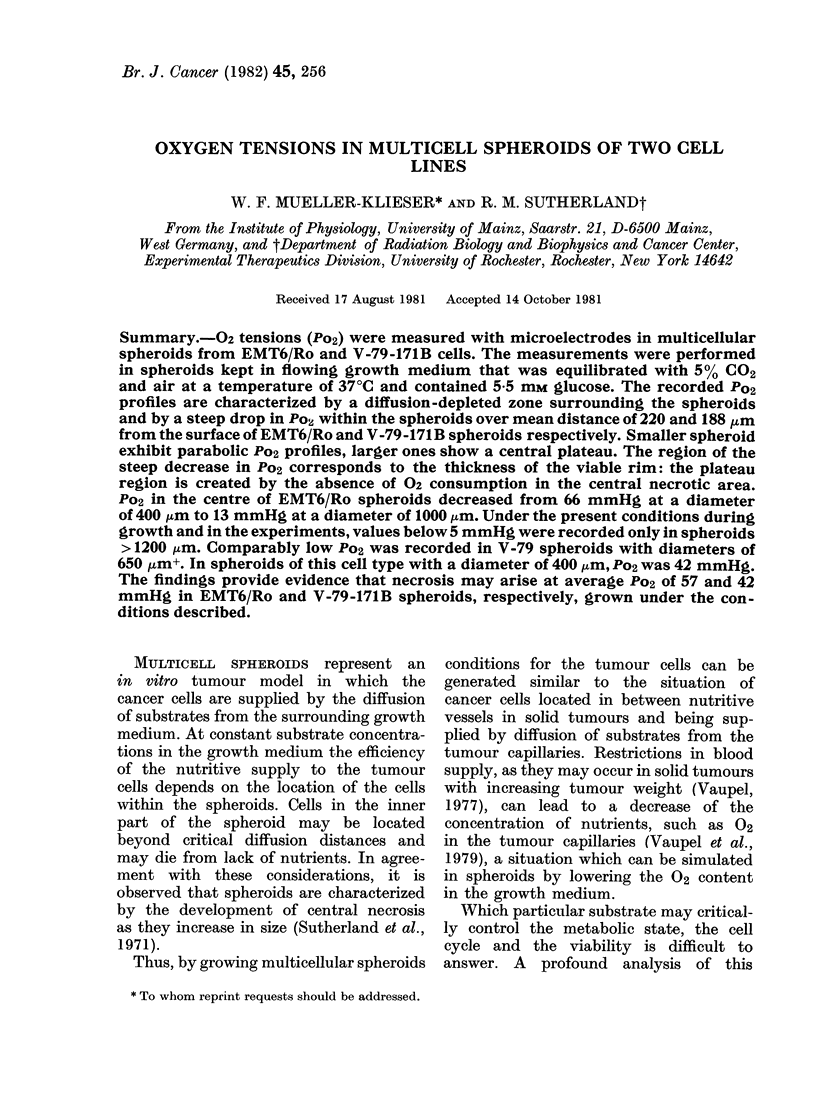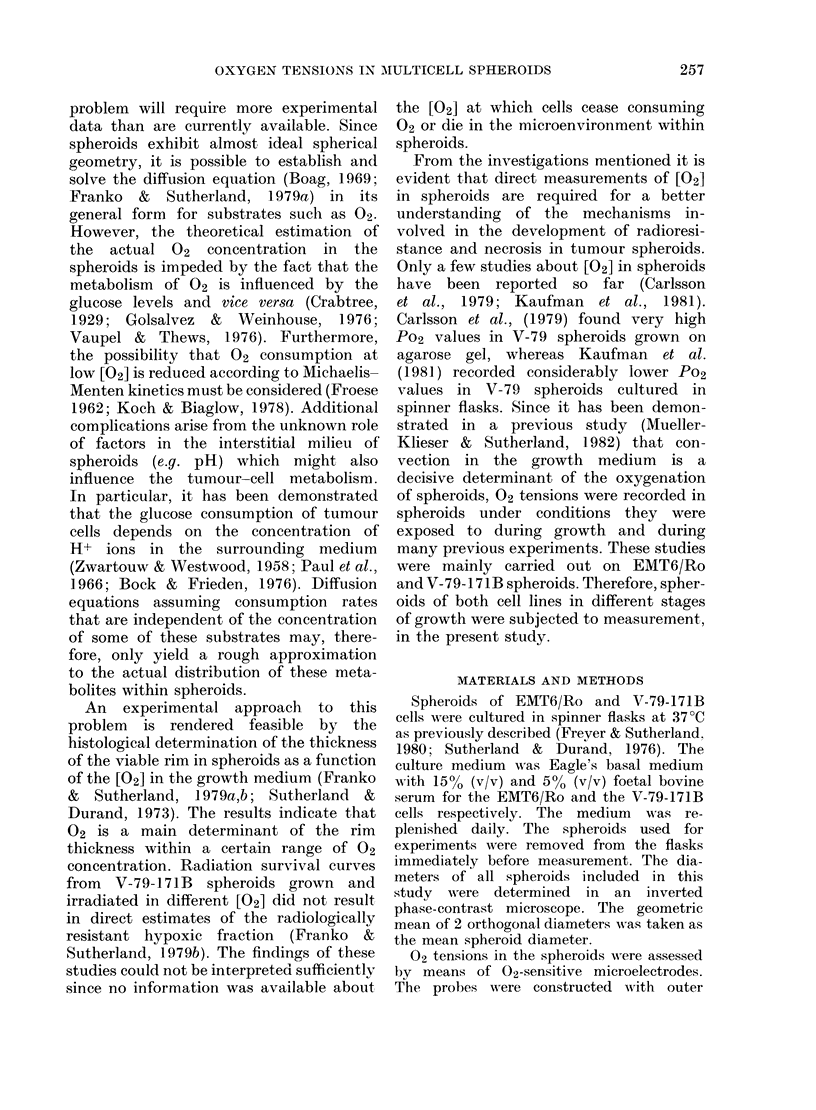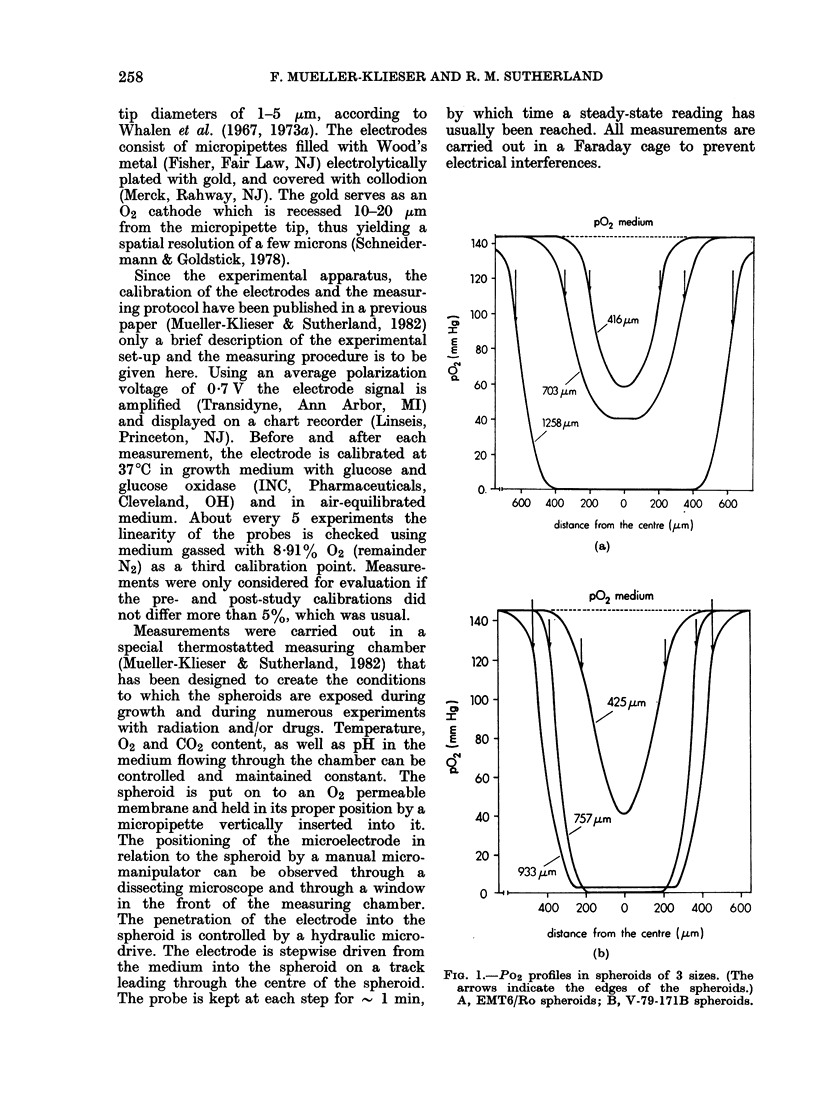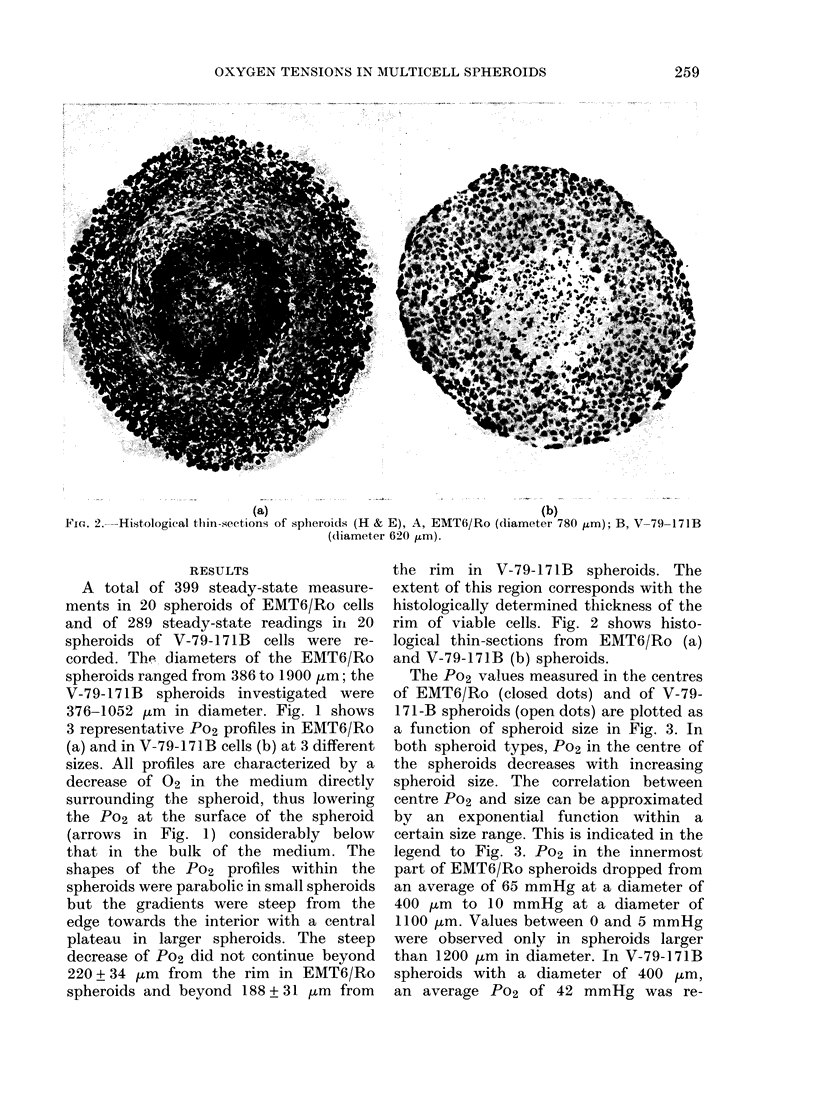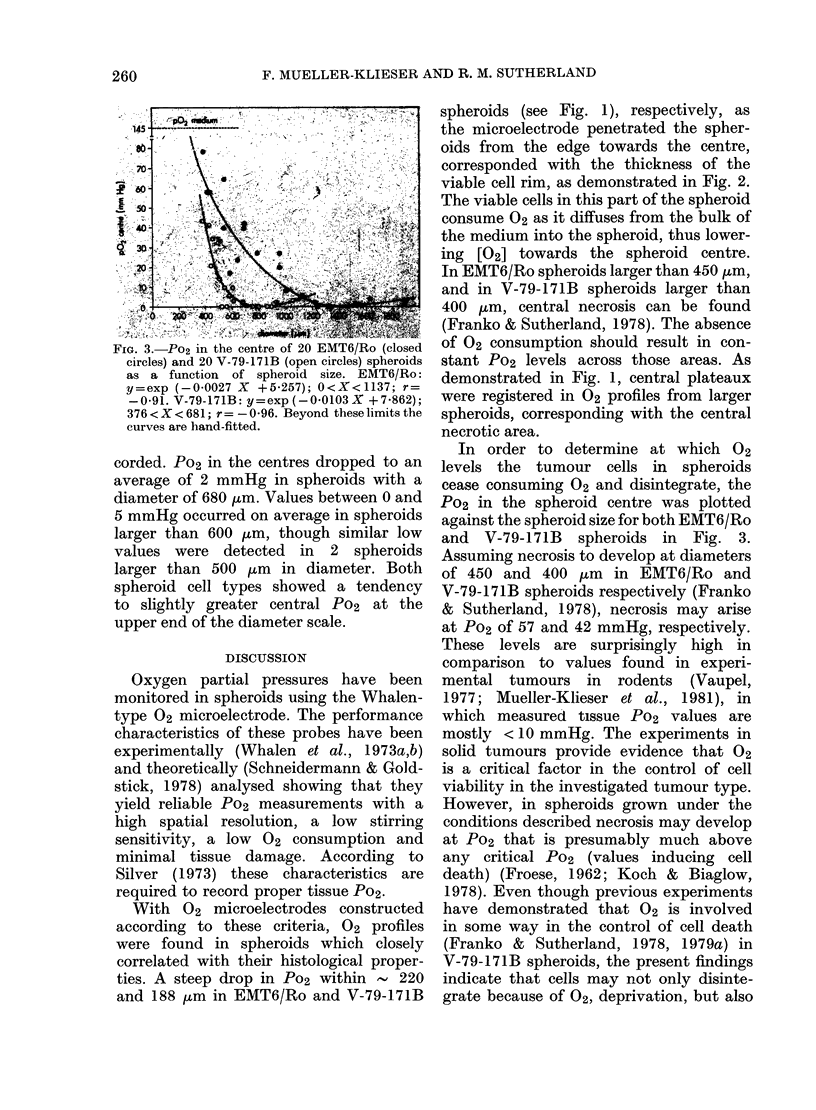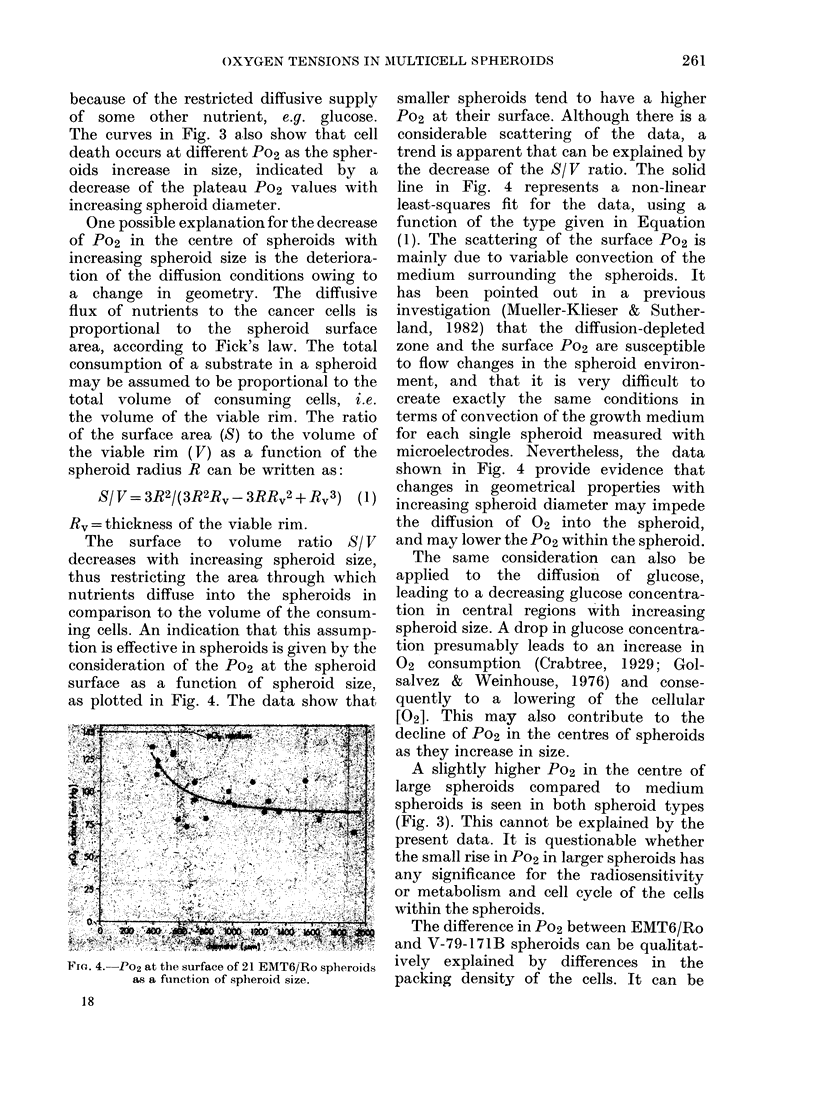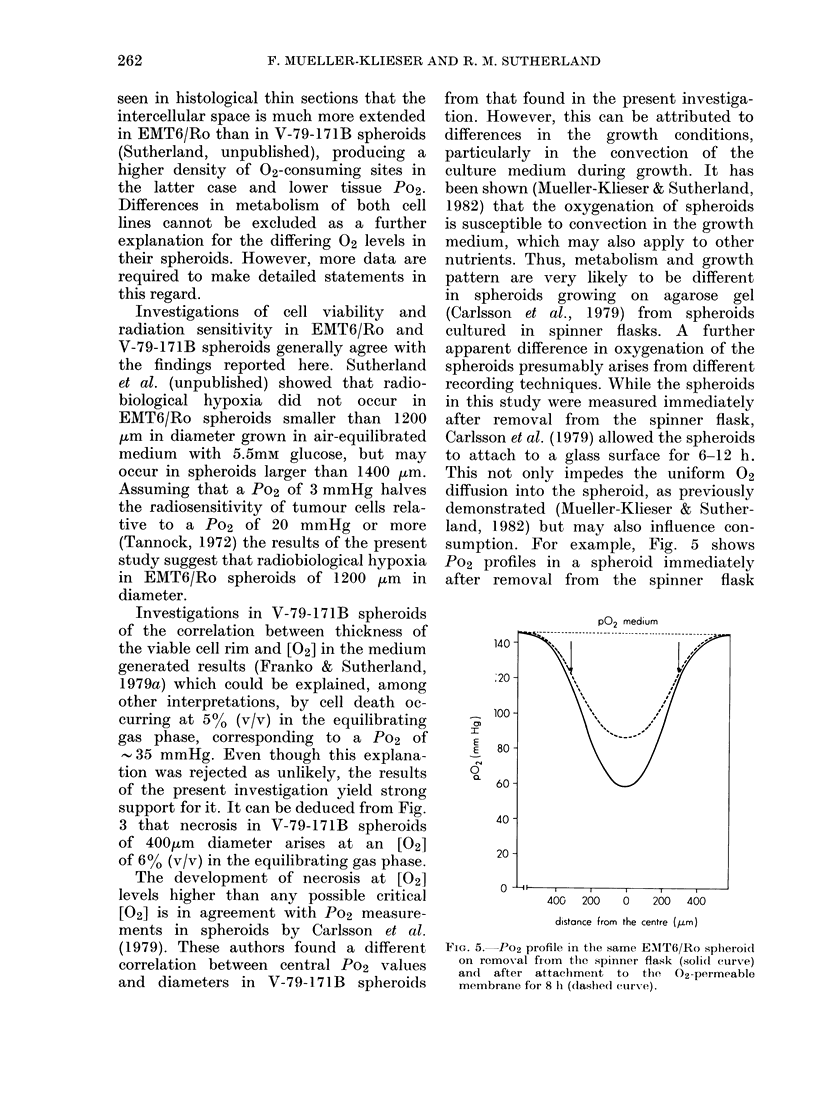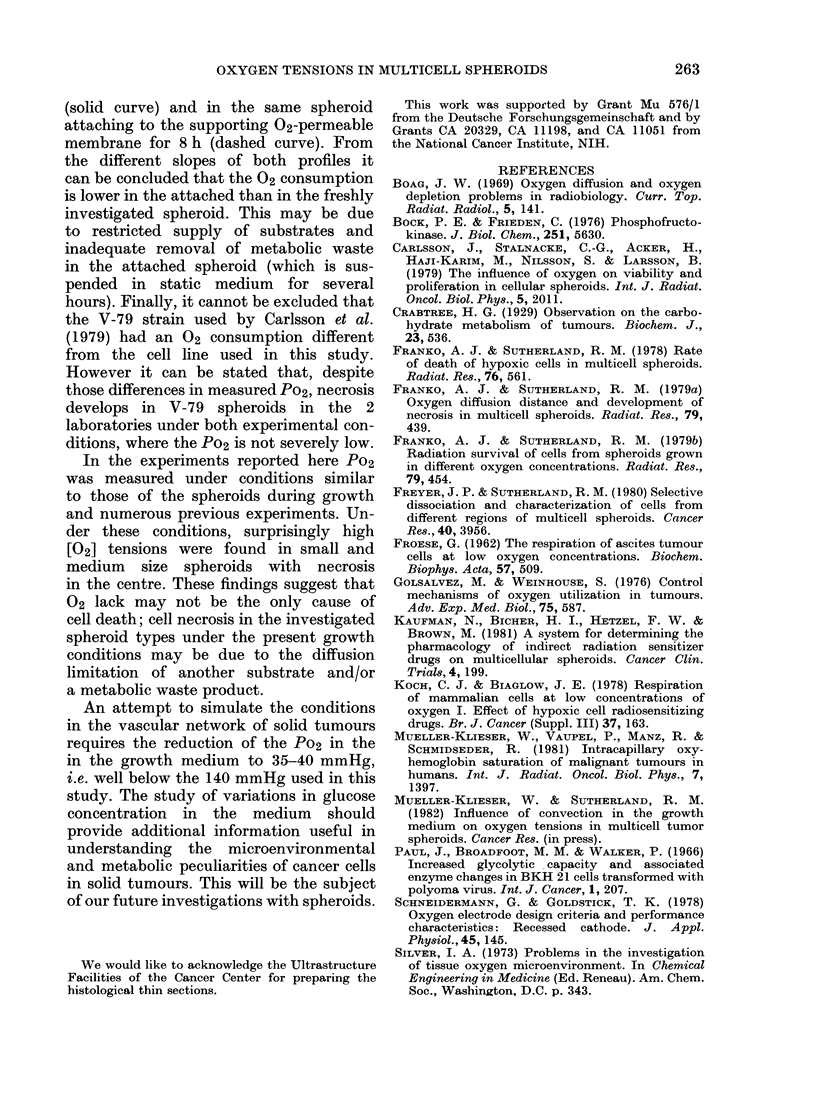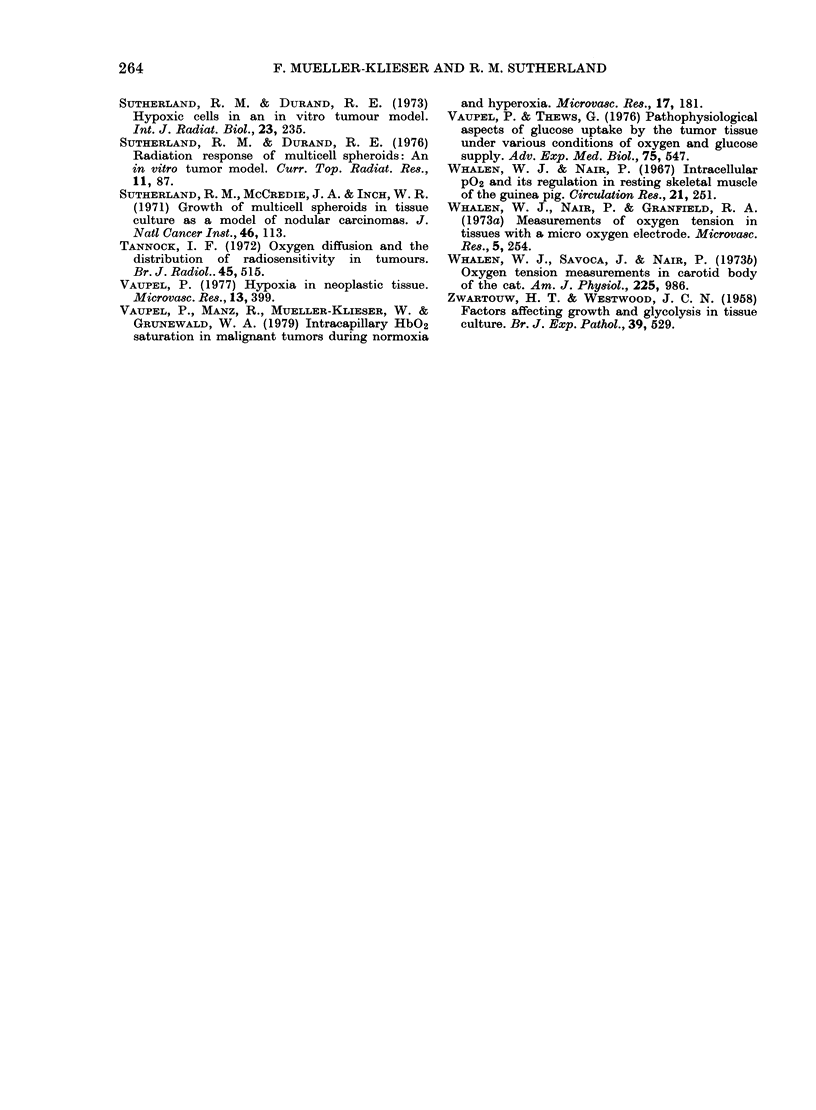# Oxygen tensions in multicell spheroids of two cell lines.

**DOI:** 10.1038/bjc.1982.41

**Published:** 1982-02

**Authors:** W. F. Mueller-Klieser, R. M. Sutherland

## Abstract

**Images:**


*Br. J. Cancer* (1982) **45**, 256

### OXYGEN TENSIONS IN MULTICELL SPHEROIDS OF TWO CELL LINES

W. F. MUELLER-KLIESER* AND R. M. SUTHERLAND†

*From the Institute of Physiology, University of Mainz, Saarstr. 21, D-6500 Mainz, West Germany, and †Department of Radiation Biology and Biophysics and Cancer Center, Experimental Therapeutics Division, University of Rochester, Rochester, New York 14642*

Received 17 August 1981 Accepted 14 October 1981

**Summary.—$O_2$ tensions ($Po_2$) were measured with microelectrodes in multicellular spheroids from EMT6/Ro and V-79-171B cells. The measurements were performed in spheroids kept in flowing growth medium that was equilibrated with 5% $CO_2$ and air at a temperature of 37°C and contained 5·5 mM glucose. The recorded $Po_2$ profiles are characterized by a diffusion-depleted zone surrounding the spheroids and by a steep drop in $Po_2$ within the spheroids over mean distance of 220 and 188 $\mu$m from the surface of EMT6/Ro and V-79-171B spheroids respectively. Smaller spheroid exhibit parabolic $Po_2$ profiles, larger ones show a central plateau. The region of the steep decrease in $Po_2$ corresponds to the thickness of the viable rim: the plateau region is created by the absence of $O_2$ consumption in the central necrotic area. $Po_2$ in the centre of EMT6/Ro spheroids decreased from 66 mmHg at a diameter of 400 $\mu$m to 13 mmHg at a diameter of 1000 $\mu$m. Under the present conditions during growth and in the experiments, values below 5 mmHg were recorded only in spheroids >1200 $\mu$m. Comparably low $Po_2$ was recorded in V-79 spheroids with diameters of 650 $\mu m^+$. In spheroids of this cell type with a diameter of 400 $\mu$m, $Po_2$ was 42 mmHg. The findings provide evidence that necrosis may arise at average $Po_2$ of 57 and 42 mmHg in EMT6/Ro and V-79-171B spheroids, respectively, grown under the conditions described.**

MULTICELL SPHEROIDS represent an *in vitro* tumour model in which the cancer cells are supplied by the diffusion of substrates from the surrounding growth medium. At constant substrate concentrations in the growth medium the efficiency of the nutritive supply to the tumour cells depends on the location of the cells within the spheroids. Cells in the inner part of the spheroid may be located beyond critical diffusion distances and may die from lack of nutrients. In agreement with these considerations, it is observed that spheroids are characterized by the development of central necrosis as they increase in size (Sutherland *et al.*, 1971).

Thus, by growing multicellular spheroids conditions for the tumour cells can be generated similar to the situation of cancer cells located in between nutritive vessels in solid tumours and being supplied by diffusion of substrates from the tumour capillaries. Restrictions in blood supply, as they may occur in solid tumours with increasing tumour weight (Vaupel, 1977), can lead to a decrease of the concentration of nutrients, such as $O_2$ in the tumour capillaries (Vaupel *et al.*, 1979), a situation which can be simulated in spheroids by lowering the $O_2$ content in the growth medium.

Which particular substrate may critically control the metabolic state, the cell cycle and the viability is difficult to answer. A profound analysis of this

* To whom reprint requests should be addressed.

problem will require more experimental data than are currently available. Since spheroids exhibit almost ideal spherical geometry, it is possible to establish and solve the diffusion equation (Boag, 1969; Franko & Sutherland, 1979*a*) in its general form for substrates such as $O_2$. However, the theoretical estimation of the actual $O_2$ concentration in the spheroids is impeded by the fact that the metabolism of $O_2$ is influenced by the glucose levels and *vice versa* (Crabtree, 1929; Golsalvez & Weinhouse, 1976; Vaupel & Thews, 1976). Furthermore, the possibility that $O_2$ consumption at low $[O_2]$ is reduced according to Michaelis–Menten kinetics must be considered (Froese 1962; Koch & Biaglow, 1978). Additional complications arise from the unknown role of factors in the interstitial milieu of spheroids (*e.g.* pH) which might also influence the tumour–cell metabolism. In particular, it has been demonstrated that the glucose consumption of tumour cells depends on the concentration of $H^+$ ions in the surrounding medium (Zwartouw & Westwood, 1958; Paul *et al.*, 1966; Bock & Frieden, 1976). Diffusion equations assuming consumption rates that are independent of the concentration of some of these substrates may, therefore, only yield a rough approximation to the actual distribution of these metabolites within spheroids.

An experimental approach to this problem is rendered feasible by the histological determination of the thickness of the viable rim in spheroids as a function of the $[O_2]$ in the growth medium (Franko & Sutherland, 1979*a*,*b*; Sutherland & Durand, 1973). The results indicate that $O_2$ is a main determinant of the rim thickness within a certain range of $O_2$ concentration. Radiation survival curves from V-79-171B spheroids grown and irradiated in different $[O_2]$ did not result in direct estimates of the radiologically resistant hypoxic fraction (Franko & Sutherland, 1979*b*). The findings of these studies could not be interpreted sufficiently since no information was available about the $[O_2]$ at which cells cease consuming $O_2$ or die in the microenvironment within spheroids.

From the investigations mentioned it is evident that direct measurements of $[O_2]$ in spheroids are required for a better understanding of the mechanisms involved in the development of radioresistance and necrosis in tumour spheroids. Only a few studies about $[O_2]$ in spheroids have been reported so far (Carlsson *et al.*, 1979; Kaufman *et al.*, 1981). Carlsson *et al.*, (1979) found very high $Po_2$ values in V-79 spheroids grown on agarose gel, whereas Kaufman *et al.* (1981) recorded considerably lower $Po_2$ values in V-79 spheroids cultured in spinner flasks. Since it has been demonstrated in a previous study (Mueller-Klieser & Sutherland, 1982) that convection in the growth medium is a decisive determinant of the oxygenation of spheroids, $O_2$ tensions were recorded in spheroids under conditions they were exposed to during growth and during many previous experiments. These studies were mainly carried out on EMT6/Ro and V-79-171B spheroids. Therefore, spheroids of both cell lines in different stages of growth were subjected to measurement, in the present study.

### MATERIALS AND METHODS

Spheroids of EMT6/Ro and V-79-171B cells were cultured in spinner flasks at 37°C as previously described (Freyer & Sutherland, 1980; Sutherland & Durand, 1976). The culture medium was Eagle's basal medium with 15% (v/v) and 5% (v/v) foetal bovine serum for the EMT6/Ro and the V-79-171B cells respectively. The medium was replenished daily. The spheroids used for experiments were removed from the flasks immediately before measurement. The diameters of all spheroids included in this study were determined in an inverted phase-contrast microscope. The geometric mean of 2 orthogonal diameters was taken as the mean spheroid diameter.

$O_2$ tensions in the spheroids were assessed by means of $O_2$-sensitive microelectrodes. The probes were constructed with outer tip diameters of 1–5 μm, according to Whalen *et al.* (1967, 1973*a*). The electrodes consist of micropipettes filled with Wood's metal (Fisher, Fair Law, NJ) electrolytically plated with gold, and covered with collodion (Merck, Rahway, NJ). The gold serves as an $O_2$ cathode which is recessed 10–20 μm from the micropipette tip, thus yielding a spatial resolution of a few microns (Schneidermann & Goldstick, 1978).

Since the experimental apparatus, the calibration of the electrodes and the measuring protocol have been published in a previous paper (Mueller-Klieser & Sutherland, 1982) only a brief description of the experimental set-up and the measuring procedure is to be given here. Using an average polarization voltage of 0·7 V the electrode signal is amplified (Transidyne, Ann Arbor, MI) and displayed on a chart recorder (Linseis, Princeton, NJ). Before and after each measurement, the electrode is calibrated at 37 °C in growth medium with glucose and glucose oxidase (INC, Pharmaceuticals, Cleveland, OH) and in air-equilibrated medium. About every 5 experiments the linearity of the probes is checked using medium gassed with 8·91% $O_2$ (remainder $N_2$) as a third calibration point. Measurements were only considered for evaluation if the pre- and post-study calibrations did not differ more than 5%, which was usual.

Measurements were carried out in a special thermostatted measuring chamber (Mueller-Klieser & Sutherland, 1982) that has been designed to create the conditions to which the spheroids are exposed during growth and during numerous experiments with radiation and/or drugs. Temperature, $O_2$ and $CO_2$ content, as well as pH in the medium flowing through the chamber can be controlled and maintained constant. The spheroid is put on to an $O_2$ permeable membrane and held in its proper position by a micropipette vertically inserted into it. The positioning of the microelectrode in relation to the spheroid by a manual micromanipulator can be observed through a dissecting microscope and through a window in the front of the measuring chamber. The penetration of the electrode into the spheroid is controlled by a hydraulic microdrive. The electrode is stepwise driven from the medium into the spheroid on a track leading through the centre of the spheroid. The probe is kept at each step for ~ 1 min, by which time a steady-state reading has usually been reached. All measurements are carried out in a Faraday cage to prevent electrical interferences.

FIG. 1.—$Po_2$ profiles in spheroids of 3 sizes. (The arrows indicate the edges of the spheroids.) A, EMT6/Ro spheroids; B, V-79-171B spheroids.

FIG. 2.—Histological thin-sections of spheroids (H & E), A, EMT6/Ro (diameter 780 $\mu$m); B, V–79–171B (diameter 620 $\mu$m).

RESULTS

A total of 399 steady-state measurements in 20 spheroids of EMT6/Ro cells and of 289 steady-state readings in 20 spheroids of V-79-171B cells were recorded. The diameters of the EMT6/Ro spheroids ranged from 386 to 1900 $\mu$m; the V-79-171B spheroids investigated were 376–1052 $\mu$m in diameter. Fig. 1 shows 3 representative $P\text{o}_2$ profiles in EMT6/Ro (a) and in V-79-171B cells (b) at 3 different sizes. All profiles are characterized by a decrease of $O_2$ in the medium directly surrounding the spheroid, thus lowering the $P\text{o}_2$ at the surface of the spheroid (arrows in Fig. 1) considerably below that in the bulk of the medium. The shapes of the $P\text{o}_2$ profiles within the spheroids were parabolic in small spheroids but the gradients were steep from the edge towards the interior with a central plateau in larger spheroids. The steep decrease of $P\text{o}_2$ did not continue beyond $220 \pm 34$ $\mu$m from the rim in EMT6/Ro spheroids and beyond $188 \pm 31$ $\mu$m from the rim in V-79-171B spheroids. The extent of this region corresponds with the histologically determined thickness of the rim of viable cells. Fig. 2 shows histological thin-sections from EMT6/Ro (a) and V-79-171B (b) spheroids.

The $P\text{o}_2$ values measured in the centres of EMT6/Ro (closed dots) and of V-79-171-B spheroids (open dots) are plotted as a function of spheroid size in Fig. 3. In both spheroid types, $P\text{o}_2$ in the centre of the spheroids decreases with increasing spheroid size. The correlation between centre $P\text{o}_2$ and size can be approximated by an exponential function within a certain size range. This is indicated in the legend to Fig. 3. $P\text{o}_2$ in the innermost part of EMT6/Ro spheroids dropped from an average of 65 mmHg at a diameter of 400 $\mu$m to 10 mmHg at a diameter of 1100 $\mu$m. Values between 0 and 5 mmHg were observed only in spheroids larger than 1200 $\mu$m in diameter. In V-79-171B spheroids with a diameter of 400 $\mu$m, an average $P\text{o}_2$ of 42 mmHg was re-

FIG. 3.—$P\text{o}_2$ in the centre of 20 EMT6/Ro (closed circles) and 20 V-79-171B (open circles) spheroids as a function of spheroid size. EMT6/Ro: $y = \exp(-0{\cdot}0027\ X + 5{\cdot}257)$; $0 < X < 1137$; $r = -0{\cdot}91$. V-79-171B: $y = \exp(-0{\cdot}0103\ X + 7{\cdot}862)$; $376 < X < 681$; $r = -0{\cdot}96$. Beyond these limits the curves are hand-fitted.

corded. $P\text{o}_2$ in the centres dropped to an average of 2 mmHg in spheroids with a diameter of 680 μm. Values between 0 and 5 mmHg occurred on average in spheroids larger than 600 μm, though similar low values were detected in 2 spheroids larger than 500 μm in diameter. Both spheroid cell types showed a tendency to slightly greater central $P\text{o}_2$ at the upper end of the diameter scale.

### DISCUSSION

Oxygen partial pressures have been monitored in spheroids using the Whalen-type $O_2$ microelectrode. The performance characteristics of these probes have been experimentally (Whalen *et al.*, 1973*a,b*) and theoretically (Schneidermann & Goldstick, 1978) analysed showing that they yield reliable $P\text{o}_2$ measurements with a high spatial resolution, a low stirring sensitivity, a low $O_2$ consumption and minimal tissue damage. According to Silver (1973) these characteristics are required to record proper tissue $P\text{o}_2$.

With $O_2$ microelectrodes constructed according to these criteria, $O_2$ profiles were found in spheroids which closely correlated with their histological properties. A steep drop in $P\text{o}_2$ within ~ 220 and 188 μm in EMT6/Ro and V-79-171B spheroids (see Fig. 1), respectively, as the microelectrode penetrated the spheroids from the edge towards the centre, corresponded with the thickness of the viable cell rim, as demonstrated in Fig. 2. The viable cells in this part of the spheroid consume $O_2$ as it diffuses from the bulk of the medium into the spheroid, thus lowering $[O_2]$ towards the spheroid centre. In EMT6/Ro spheroids larger than 450 μm, and in V-79-171B spheroids larger than 400 μm, central necrosis can be found (Franko & Sutherland, 1978). The absence of $O_2$ consumption should result in constant $P\text{o}_2$ levels across those areas. As demonstrated in Fig. 1, central plateaux were registered in $O_2$ profiles from larger spheroids, corresponding with the central necrotic area.

In order to determine at which $O_2$ levels the tumour cells in spheroids cease consuming $O_2$ and disintegrate, the $P\text{o}_2$ in the spheroid centre was plotted against the spheroid size for both EMT6/Ro and V-79-171B spheroids in Fig. 3. Assuming necrosis to develop at diameters of 450 and 400 μm in EMT6/Ro and V-79-171B spheroids respectively (Franko & Sutherland, 1978), necrosis may arise at $P\text{o}_2$ of 57 and 42 mmHg, respectively. These levels are surprisingly high in comparison to values found in experimental tumours in rodents (Vaupel, 1977; Mueller-Klieser *et al.*, 1981), in which measured tissue $P\text{o}_2$ values are mostly $< 10$ mmHg. The experiments in solid tumours provide evidence that $O_2$ is a critical factor in the control of cell viability in the investigated tumour type. However, in spheroids grown under the conditions described necrosis may develop at $P\text{o}_2$ that is presumably much above any critical $P\text{o}_2$ (values inducing cell death) (Froese, 1962; Koch & Biaglow, 1978). Even though previous experiments have demonstrated that $O_2$ is involved in some way in the control of cell death (Franko & Sutherland, 1978, 1979*a*) in V-79-171B spheroids, the present findings indicate that cells may not only disintegrate because of $O_2$, deprivation, but also because of the restricted diffusive supply of some other nutrient, *e.g.* glucose. The curves in Fig. 3 also show that cell death occurs at different $Po_2$ as the spheroids increase in size, indicated by a decrease of the plateau $Po_2$ values with increasing spheroid diameter.

One possible explanation for the decrease of $Po_2$ in the centre of spheroids with increasing spheroid size is the deterioration of the diffusion conditions owing to a change in geometry. The diffusive flux of nutrients to the cancer cells is proportional to the spheroid surface area, according to Fick's law. The total consumption of a substrate in a spheroid may be assumed to be proportional to the total volume of consuming cells, *i.e.* the volume of the viable rim. The ratio of the surface area ($S$) to the volume of the viable rim ($V$) as a function of the spheroid radius $R$ can be written as:

$$S/V = 3R^2/(3R^2R_V - 3RR_V^2 + R_V^3) \quad (1)$$

$R_V$ = thickness of the viable rim.

The surface to volume ratio $S/V$ decreases with increasing spheroid size, thus restricting the area through which nutrients diffuse into the spheroids in comparison to the volume of the consuming cells. An indication that this assumption is effective in spheroids is given by the consideration of the $Po_2$ at the spheroid surface as a function of spheroid size, as plotted in Fig. 4. The data show that

FIG. 4.—$Po_2$ at the surface of 21 EMT6/Ro spheroids as a function of spheroid size.

smaller spheroids tend to have a higher $Po_2$ at their surface. Although there is a considerable scattering of the data, a trend is apparent that can be explained by the decrease of the $S/V$ ratio. The solid line in Fig. 4 represents a non-linear least-squares fit for the data, using a function of the type given in Equation (1). The scattering of the surface $Po_2$ is mainly due to variable convection of the medium surrounding the spheroids. It has been pointed out in a previous investigation (Mueller-Klieser & Sutherland, 1982) that the diffusion-depleted zone and the surface $Po_2$ are susceptible to flow changes in the spheroid environment, and that it is very difficult to create exactly the same conditions in terms of convection of the growth medium for each single spheroid measured with microelectrodes. Nevertheless, the data shown in Fig. 4 provide evidence that changes in geometrical properties with increasing spheroid diameter may impede the diffusion of $O_2$ into the spheroid, and may lower the $Po_2$ within the spheroid.

The same consideration can also be applied to the diffusion of glucose, leading to a decreasing glucose concentration in central regions with increasing spheroid size. A drop in glucose concentration presumably leads to an increase in $O_2$ consumption (Crabtree, 1929; Golsalvez & Weinhouse, 1976) and consequently to a lowering of the cellular [$O_2$]. This may also contribute to the decline of $Po_2$ in the centres of spheroids as they increase in size.

A slightly higher $Po_2$ in the centre of large spheroids compared to medium spheroids is seen in both spheroid types (Fig. 3). This cannot be explained by the present data. It is questionable whether the small rise in $Po_2$ in larger spheroids has any significance for the radiosensitivity or metabolism and cell cycle of the cells within the spheroids.

The difference in $Po_2$ between EMT6/Ro and V-79-171B spheroids can be qualitatively explained by differences in the packing density of the cells. It can be seen in histological thin sections that the intercellular space is much more extended in EMT6/Ro than in V-79-171B spheroids (Sutherland, unpublished), producing a higher density of $O_2$-consuming sites in the latter case and lower tissue $Po_2$. Differences in metabolism of both cell lines cannot be excluded as a further explanation for the differing $O_2$ levels in their spheroids. However, more data are required to make detailed statements in this regard.

Investigations of cell viability and radiation sensitivity in EMT6/Ro and V-79-171B spheroids generally agree with the findings reported here. Sutherland *et al.* (unpublished) showed that radiobiological hypoxia did not occur in EMT6/Ro spheroids smaller than 1200 $\mu$m in diameter grown in air-equilibrated medium with 5.5mM glucose, but may occur in spheroids larger than 1400 $\mu$m. Assuming that a $Po_2$ of 3 mmHg halves the radiosensitivity of tumour cells relative to a $Po_2$ of 20 mmHg or more (Tannock, 1972) the results of the present study suggest that radiobiological hypoxia in EMT6/Ro spheroids of 1200 $\mu$m in diameter.

Investigations in V-79-171B spheroids of the correlation between thickness of the viable cell rim and $[O_2]$ in the medium generated results (Franko & Sutherland, 1979*a*) which could be explained, among other interpretations, by cell death occurring at 5% (v/v) in the equilibrating gas phase, corresponding to a $Po_2$ of $\sim$35 mmHg. Even though this explanation was rejected as unlikely, the results of the present investigation yield strong support for it. It can be deduced from Fig. 3 that necrosis in V-79-171B spheroids of 400$\mu$m diameter arises at an $[O_2]$ of 6% (v/v) in the equilibrating gas phase.

The development of necrosis at $[O_2]$ levels higher than any possible critical $[O_2]$ is in agreement with $Po_2$ measurements in spheroids by Carlsson *et al.* (1979). These authors found a different correlation between central $Po_2$ values and diameters in V-79-171B spheroids from that found in the present investigation. However, this can be attributed to differences in the growth conditions, particularly in the convection of the culture medium during growth. It has been shown (Mueller-Klieser & Sutherland, 1982) that the oxygenation of spheroids is susceptible to convection in the growth medium, which may also apply to other nutrients. Thus, metabolism and growth pattern are very likely to be different in spheroids growing on agarose gel (Carlsson *et al.*, 1979) from spheroids cultured in spinner flasks. A further apparent difference in oxygenation of the spheroids presumably arises from different recording techniques. While the spheroids in this study were measured immediately after removal from the spinner flask, Carlsson *et al.* (1979) allowed the spheroids to attach to a glass surface for 6–12 h. This not only impedes the uniform $O_2$ diffusion into the spheroid, as previously demonstrated (Mueller-Klieser & Sutherland, 1982) but may also influence consumption. For example, Fig. 5 shows $Po_2$ profiles in a spheroid immediately after removal from the spinner flask

FIG. 5.—$Po_2$ profile in the same EMT6/Ro spheroid on removal from the spinner flask (solid curve) and after attachment to the $O_2$-permeable membrane for 8 h (dashed curve).

(solid curve) and in the same spheroid attaching to the supporting $O_2$-permeable membrane for 8 h (dashed curve). From the different slopes of both profiles it can be concluded that the $O_2$ consumption is lower in the attached than in the freshly investigated spheroid. This may be due to restricted supply of substrates and inadequate removal of metabolic waste in the attached spheroid (which is suspended in static medium for several hours). Finally, it cannot be excluded that the V-79 strain used by Carlsson *et al.* (1979) had an $O_2$ consumption different from the cell line used in this study. However it can be stated that, despite those differences in measured $Po_2$, necrosis develops in V-79 spheroids in the 2 laboratories under both experimental conditions, where the $Po_2$ is not severely low.

In the experiments reported here $Po_2$ was measured under conditions similar to those of the spheroids during growth and numerous previous experiments. Under these conditions, surprisingly high $[O_2]$ tensions were found in small and medium size spheroids with necrosis in the centre. These findings suggest that $O_2$ lack may not be the only cause of cell death; cell necrosis in the investigated spheroid types under the present growth conditions may be due to the diffusion limitation of another substrate and/or a metabolic waste product.

An attempt to simulate the conditions in the vascular network of solid tumours requires the reduction of the $Po_2$ in the in the growth medium to 35–40 mmHg, *i.e.* well below the 140 mmHg used in this study. The study of variations in glucose concentration in the medium should provide additional information useful in understanding the microenvironmental and metabolic peculiarities of cancer cells in solid tumours. This will be the subject of our future investigations with spheroids.

We would like to acknowledge the Ultrastructure Facilities of the Cancer Center for preparing the histological thin sections.

This work was supported by Grant Mu 576/1 from the Deutsche Forschungsgemeinschaft and by Grants CA 20329, CA 11198, and CA 11051 from the National Cancer Institute, NIH.

### REFERENCES

Boag, J. W. (1969) Oxygen diffusion and oxygen depletion problems in radiobiology. *Curr. Top. Radiat. Radiol.*, **5**, 141.

Bock, P. E. & Frieden, C. (1976) Phosphofructokinase. *J. Biol. Chem.*, **251**, 5630.

Carlsson, J., Stalnacke, C.-G., Acker, H., Haji-Karim, M., Nilsson, S. & Larsson, B. (1979) The influence of oxygen on viability and proliferation in cellular spheroids. *Int. J. Radiat. Oncol. Biol. Phys.*, **5**, 2011.

Crabtree, H. G. (1929) Observation on the carbohydrate metabolism of tumours. *Biochem. J.*, **23**, 536.

Franko, A. J. & Sutherland, R. M. (1978) Rate of death of hypoxic cells in multicell spheroids. *Radiat. Res.*, **76**, 561.

Franko, A. J. & Sutherland, R. M. (1979*a*) Oxygen diffusion distance and development of necrosis in multicell spheroids. *Radiat. Res.*, **79**, 439.

Franko, A. J. & Sutherland, R. M. (1979*b*) Radiation survival of cells from spheroids grown in different oxygen concentrations. *Radiat. Res.*, **79**, 454.

Freyer, J. P. & Sutherland, R. M. (1980) Selective dissociation and characterization of cells from different regions of multicell spheroids. *Cancer Res.*, **40**, 3956.

Froese, G. (1962) The respiration of ascites tumour cells at low oxygen concentrations. *Biochem. Biophys. Acta*, **57**, 509.

Golsalvez, M. & Weinhouse, S. (1976) Control mechanisms of oxygen utilization in tumours. *Adv. Exp. Med. Biol.*, **75**, 587.

Kaufman, N., Bicher, H. I., Hetzel, F. W. & Brown, M. (1981) A system for determining the pharmacology of indirect radiation sensitizer drugs on multicellular spheroids. *Cancer Clin. Trials*, **4**, 199.

Koch, C. J. & Biaglow, J. E. (1978) Respiration of mammalian cells at low concentrations of oxygen I. Effect of hypoxic cell radiosensitizing drugs. *Br. J. Cancer* (Suppl. III) **37**, 163.

Mueller-Klieser, W., Vaupel, P., Manz, R. & Schmidseder, R. (1981) Intracapillary oxyhemoglobin saturation of malignant tumours in humans. *Int. J. Radiat. Oncol. Biol. Phys.*, **7**, 1397.

Mueller-Klieser, W. & Sutherland, R. M. (1982) Influence of convection in the growth medium on oxygen tensions in multicell tumor spheroids. *Cancer Res.* (in press).

Paul, J., Broadfoot, M. M. & Walker, P. (1966) Increased glycolytic capacity and associated enzyme changes in BKH 21 cells transformed with polyoma virus. *Int. J. Cancer*, **1**, 207.

Schneidermann, G. & Goldstick, T. K. (1978) Oxygen electrode design criteria and performance characteristics: Recessed cathode. *J. Appl. Physiol.*, **45**, 145.

Silver, I. A. (1973) Problems in the investigation of tissue oxygen microenvironment. In *Chemical Engineering in Medicine* (Ed. Reneau). Am. Chem. Soc., Washington, D.C. p. 343.

SUTHERLAND, R. M. & DURAND, R. E. (1973) Hypoxic cells in an in vitro tumour model. *Int. J. Radiat. Biol.*, **23**, 235.

SUTHERLAND, R. M. & DURAND, R. E. (1976) Radiation response of multicell spheroids: An *in vitro* tumor model. *Curr. Top. Radiat. Res.*, **11**, 87.

SUTHERLAND, R. M., MCCREDIE, J. A. & INCH, W. R. (1971) Growth of multicell spheroids in tissue culture as a model of nodular carcinomas. *J. Natl Cancer Inst.*, **46**, 113.

TANNOCK, I. F. (1972) Oxygen diffusion and the distribution of radiosensitivity in tumours. *Br. J. Radiol.*. **45**, 515.

VAUPEL, P. (1977) Hypoxia in neoplastic tissue. *Microvasc. Res.*, **13**, 399.

VAUPEL, P., MANZ, R., MUELLER-KLIESER, W. & GRUNEWALD, W. A. (1979) Intracapillary $HbO_2$ saturation in malignant tumors during normoxia and hyperoxia. *Microvasc. Res.*, **17**, 181.

VAUPEL, P. & THEWS, G. (1976) Pathophysiological aspects of glucose uptake by the tumor tissue under various conditions of oxygen and glucose supply. *Adv. Exp. Med. Biol.*, **75**, 547.

WHALEN, W. J. & NAIR, P. (1967) Intracellular $pO_2$ and its regulation in resting skeletal muscle of the guinea pig. *Circulation Res.*, **21**, 251.

WHALEN, W. J., NAIR, P. & GRANFIELD, R. A. (1973*a*) Measurements of oxygen tension in tissues with a micro oxygen electrode. *Microvasc. Res.*, **5**, 254.

WHALEN, W. J., SAVOCA, J. & NAIR, P. (1973*b*) Oxygen tension measurements in carotid body of the cat. *Am. J. Physiol.*, **225**, 986.

ZWARTOUW, H. T. & WESTWOOD, J. C. N. (1958) Factors affecting growth and glycolysis in tissue culture. *Br. J. Exp. Pathol.*, **39**, 529.